# Ocular fundus pathology and chronic kidney disease in a Chinese population

**DOI:** 10.1186/1471-2369-12-62

**Published:** 2011-11-17

**Authors:** Bixia Gao, Ling Zhu, Yingzi Pan, Shuwen Yang, Luxia Zhang, Haiyan Wang

**Affiliations:** 1 Renal Division, Department of Medicine, Peking University First Hospital; Peking University Institute of Nephrology, Key Laboratory of Renal Disease, Ministry of Health of China; Key Laboratory of Chronic Kidney Disease Prevention and Treatment (Peking University), Ministry of Education, China; 2 Physical Examination Center, Beijing Hospital; Beijing, China; 3 Division of Ophthalmology, Peking University First Hospital; Beijing, China

## Abstract

**Background:**

Previous study indicated a high prevalence of ocular fundus pathology among patients with chronic kidney disease (CKD), while the relationship between them has never been explored in a Chinese Population.

**Methods:**

This cross-sectional study included 9 670 participants enrolled in a medical screening program. Ocular fundus examination was performed by ophthalmologists using ophthalmoscopes. The presence of eGFR less than 60 mL/min/1.73 m^2 ^and/or proteinuria was defined as CKD.

**Results:**

Compared to participants without CKD, participants with CKD had higher prevalence of retinopathy (28.5% vs. 16.3%, P < 0.001), glaucoma suspect (3.1% vs. 1.8%, P = 0.004), age-related macular degeneration (1.7% vs. 0.9%, P = 0.01) and overall eye pathology (32.0% vs. 19.4%, P < 0.001). After adjusting for potential confounders, the odds ratio of proteinuria for overall eye pathology and retinopathy was 1.29 (95% confidence interval [CI] 1.07-1.55) and 1.37 (95% CI 1.12-1.67), respectively. The results were robust after excluding participants with hypertension or with diabetes.

**Conclusions:**

Ocular fundus pathology is common among Chinese patients with CKD. Regular eye exam among persons with proteinuria is warranted.

## Background

Chronic kidney disease (CKD) is growing as a global public health problem: it affects 10-16% of the adult population in Asia, Australia, Europe and the United States [1]. CKD has been associated with other chronic conditions, including cardiovascular disease [2], cancer [3] and cognitive function [4]. Recently, several types of ocular fundus pathology has been associated with CKD, such as retinal microvascular abnormalities [3,5,6], age-related macular degeneration [7-9], and increased intraocular pressure [10]. A recent study among 1 904 CKD patients in the United States indicated the overall prevalence of ocular fundus pathology among CKD patients was as high as 45% [11], suggesting the necessity of regular eye examination among those patients. The prevalence of eye pathology varied with races, indicating effects of genetic and socioeconomic differences [11]. To the best our knowledge, the relationship between ocular fundus pathology and CKD has never been explored in a Chinese Population.

Therefore, we investigate the cross-sectional association between ocular fundus pathology and CKD among 9 670 Chinese participants in a standard medical screening program.

## Methods

### Study Population

From 2007-2010, there were 10 341 participants enrolled in a medical screening program run by the Health Examination Center of Beijing Hospital. Those participants come from all over Beijing to receive a regular paid health examination. Among them, 9 644 (93.3%) had results of ocular fundus examination and were therefore included in the analysis. The following participants did not agree to receive the examination and were therefore excluded from the present analysis. This study was approved by the institutional review board at Beijing Hospital. All of the participants gave their written informed consent to participate in the study.

### Evaluation of ocular fundus pathology

Ocular fundus examination was performed by 3 experienced ophthalmologists, using ophthalmoscopes (Welch Allyn, NY, US) without pharmacologic compounds to achieve pupillary dilation. Results of fundus examination was classified as: 1) retinopathy defined as vascular pathology as a result of diabetes, hypertension or other conditions [12,13]. The presence of retinal microaneurysms only, blot and/or flame hemorrhages only, hemorrhages and/or microaneurysms, cotton-wool spots, hard exudates, intraretinal microvascular abnormalities, venous beading, arteriovenous nicking, new vessels on the disc and elsewhere, and preretinal and vitreous hemorrhages was defined as retinopathy. Arteriolar narrowing and arteriovenous nicking were also defined as retinopathy. 2) glaucoma suspect defined as cup-to-disc ratio of 0.6 or higher; 3) age-related macular degeneration suggested by large drusen and pigmentary changes; 4) other fundus pathology, such as other macular abnormalities and optic nerve atrophy. "Any ocular fundus pathology" was defined by the presence of at least one of fundus abnormalities mentioned above.

### Evaluation of kidney damage indicators

Blood was collected after an overnight fast of at least 10 hours. Serum creatinine was measured by enzymatic method on an Olympus AU5400 (Olympus, Tokyo, Japan). Estimated glomerular filtration rate (eGFR) was calculated using the Chronic Kidney Disease Epidemiology Collaboration (CKD-EPI) equation [14]. Reduced renal function was defined as an eGFR less than 60 mL/min/1.73 m^2^. Proteinuria was measured on a morning urine sample using urinary dipstick test. Participants with pyuria were excluded from the analysis of proteinuria due to concern of urinary tract infection; women during menstruation were asked to receive urine routine test 3 days after menstruation. A dipstick result of trace urine protein or more was defined as proteinuria. The presence of eGFR less than 60 mL/min/1.73 m^2 ^and/or proteinuria was defined as CKD.

### Evaluation of other conditions

History of cardiovascular disease (CVD) was self-reported. All participants were asked, "Have you ever been told by a doctor that you had a heart attack?" and, "Have you ever been told by a doctor that you had a stroke?". Blood pressure was measured after resting for at least 5 minutes. Hypertension was defined as systolic blood pressure (BP) ≥140 mmHg or diastolic BP ≥90 mmHg, or self-reported history of hypertension. Fasting blood glucose was measured enzymatically with a glucose oxidase method using the Olympus AU5400 (Olympus, Tokyo, Japan). Diabetes was defined as fasting plasma glucose ≥126mg/dL (7.0 mmol/L), or self-reported history of diabetes.

Serum low-density lipoprotein (LDL)-cholesterol, high-density lipoprotein (HDL)-cholesterol, triglycerides and uric acid were determined with an Olympus AU5400 (Olympus, Tokyo, Japan).

### Statistical analyses

Characteristics of participants were reported as mean ± standard deviation for continuous variables and as percentage for binary variables. Comparisons between those with and without fundus pathology (or retinopathy) were made using t test or Wilcoxon rank-sum test for continuous variables and Chi-square test for discrete variables.

The association between fundus pathology (or retinopathy) was analyzed using Logistic regression models. We analyzed eGFR as categorical variable: ≥ 90 mL/min/1.73m^2 ^(reference), 60-89 mL/min/1.73m^2 ^and <60 mL/min/1.73m^2^. Multivariable models were constructed to adjust for potential confounding variables, including age (continuous), sex, BMI (<18.5, 18.5-24.9, 25.0-27.0, ≥27.0; kg/m^2^), hypertension (yes/no), diabetes (yes/no), history of CVD (yes/no)), plasma uric acid (continuous). Crude and adjusted odds ratios (ORs) with 95% confidence interval (CI) were reported.

In our secondary analysis, participants with diabetes or/and hypertension were excluded. Then the same Logistic regression model was build to examine the association between fundus pathology (or retinopathy) and CKD.

All P values are 2-tailed. Statistical tests were performed using SPSS statistical package, version 10.0 (SPSS, Inc., Chicago, IL)

## Results

The average age of 9 644 participants was 52.8 ± 16.0 years (range 25-99 years), and 61.6% of them were males. The percentage of eGFR ≥90 mL/min/1.73m^2^, 60-89 mL/min/1.73m^2 ^and <60 mL/min/1.73m^2 ^was 61.7%, 34.5% and 3.8%, respectively. The prevalence of proteinuria was 11.2%; the overall prevalence of CKD was 13.9%.

Characteristics of the participants stratified by ocular fundus pathology were shown in Table 1. Participants with ocular fundus pathology were older, had higher percentage of males, hypertension, diabetes and history of CVD compared to participants without ocular fundus pathology; participants with retinopathy had the similar pattern of characteristics.

**Table 1:** Characteristics of the participants by status of ocular fundus examination.

	Any ocular fundus pathology			Retinopathy	
	
	Yes (n = 2037)	No (n = 7607)	P value	Yes (n = 1670)	P value*
Age (year)	67.9 ± 11.4	48.8 ± 14.7	<0.001	69.2 ± 9.9	<0.001
Male (%)	67.2	59.8	<0.001	68.3	<0.001
BMI (kg/m^2^)	25.0 ± 3.4	24.3 ± 3.4	<0.001	25.2 ± 3.4	<0.001
Hypertension (%)	60.0	23.3	<0.001	63.8	<0.001
Diabetes (%)	20.2	7.3	<0.001	21.5	<0.001
Cardiovascular disease (%)	14.4	3.3	<0.001	15.7	<0.001
LDL-cholesterol (mg/dL)	3.0 ± 0.8	2.9 ± 0.8	<0.001	3.0 ± 0.8	<0.001
HDL-cholesterol (mg/dL)	1.4 ± 0.3	1.3 ± 0.3	0.56	1.34 ± 0.3	0.57
Triglycerides (mg/dL)	1.6 ± 1.1	1.6 ± 1.3	0.02	1.7 ± 1.0	0.02
Plasma UA (mg/dL)	5.8 ± 1.5	5.5 ± 1.5	<0.001	5.8 ± 1.5	<0.001
eGFR (mL/min/1.73m^2^)	81.9 ± 15.3	98.0 ± 16.2	<0.001	81.0 ± 14.6	<0.001
eGFR<60 mL/min/1.73m^2 ^(%)	9.1	2.3	<0.001	9.0	<0.001
Proteinuria (%)	14.8	10.2	<0.001	15.6	<0.001
Chronic kidney disease (%)	21.0	11.9	<0.001	21.7	<0.001

* Compared with participants without any ocular fundus pathology.

BMI, body mass index; LDL, low-density lipoprotein; HDL, high-density lipoprotein; UA, uric acid; eGFR, estimated glomerular filtration rate.

Among all participants, the prevalence of retinopathy, glaucoma suspect, age-related macular degeneration and other ocular fundus pathology was 17.3%, 2.0%, 1.0% and 1.7%, respectively. The overall prevalence of any ocular fundus pathology was 21.1%. There are 808 participants with hypertensive retinopathy and without diabetes, with mean systolic and diastolic BP of 137.3 ± 17.5 mmHg and 76.8 ± 10.4 mmHg, respectively. Compared to participants without CKD, participants with CKD had higher prevalence of retinopathy (28.5% vs. 16.3%, P < 0.001), glaucoma suspect (3.1% vs. 1.8%, P = 0.004), age-related macular degeneration (1.7% vs. 0.9%, P = 0.01) and overall eye pathology (32.0% vs. 19.4%, P < 0.001).

In crude analysis, eGFR was positively associated with ocular pathology (Table 2). After adjusting for other covariates, eGFR was no longer statistically associated with ocular pathology. Analysis of retinopathy produced similar results. The crude OR of proteinuria for ocular pathology was 1.53 (95% CI 1.33-1.77). After adjusting for potential confounders, the OR was attenuated to 1.29 (95% CI 1.07-1.55). The multivariable adjusted OR for retinopathy of proteinuria was 1.37 (95% CI 1.12-1.67). The adjusted OR of CKD for any ocular fundus pathology and retinopathy was 1.18 (95%CI 0.99-1.41) and 1.19 (95%CI 0.98-1.44).

**Table 2:** The association between indicators of kidney damage with ocular fundus pathology.

	eGFR ((mL/min/1.73m^2^)			Proteinuria	CKD
	≥90	60-89	<60		
Any ocular fundus pathology					
Crude OR (95% CI)	Reference	5.18 (4.64-5.77)	9.12 (7.30-11.38)	1.53 (1.33-1.77)	1.96 (1.73-2.23)
Age-, sex- adjusted OR (95% CI)	Reference	1.03 (0.89-1.19)	1.26 (0.96-1.65)	1.45 (1.22-1.73)	1.36 (1.17-1.59)
Fully adjusted OR (95% CI)*	Reference	0.97 (0.84-1.13)	1.07 (0.79-1.45)	1.29 (1.07-1.55)	1.18 (0.99-1.41)
Retinopathy					
Crude OR (95% CI)	Reference	5.87 (5.20-6.62)	7.76 (7.77-12.45)	1.63 (1.40-1.90)	2.05 (1.79-2.34)
Age-, sex- adjusted OR (95% CI)	Reference	0.96 (0.82-1.12)	1.05 (0.78-1.40)	1.55 (1.28-1.87)	1.38 (1.17-1.64)
Fully adjusted OR (95% CI)*	Reference	0.91 (0.77-1.07)	0.92 (0.66-1.27)	1.37 (1.12-1.67)	1.19 (0.98-1.44)

* Covariates included in the fully adjusted models were: age (continuous), sex, BMI (<18.5, 18.5-24.9, 25.0-27.0, ≥27.0; kg/m^2^), hypertension (yes/no), diabetes (yes/no), history of CVD (yes/no)), plasma uric acid (continuous).

The number of participants with any ocular fundus pathology and retinopathy was 2037 and 1670, respectively.

eGFR: estimated glomerular filtration rate; CKD, chronic kidney disease; OR, odds ratio; CI, confidence interval.

In our secondary analysis, among 6 248 participants without hypertension and without diabetes, the adjusted OR of proteinuria was 1.52 (95% CI 1.06-2.19) for ocular fundus pathology, and was 1.68 (95% CI 1.10-2.57) for retinopathy. eGFR was not independently associated any fundus pathology or with retinopathy in secondary analysis.

## Discussion

In this cross-sectional study of a Chinese population, the prevalence of overall ocular fundus pathology was found to be 32.0% among participants with CKD, and was significantly higher than that of participants without CKD.

Several types of ocular fundus pathology have been associated with CKD; one of the best studied is the association between retinal microvascular abnormalities and CKD. In a cross-sectional study from Singapore [3], both retinal arteriolar diameters and retinopathy was found to be independently associated with presence of CKD. The prospective analysis from the Atherosclerosis Risk in Communities Study [6] and the Cardiovascular Health Study [5] suggested that retinal microvascular abnormalities was associated with deteriorating renal function. The possible explanation for the retinopathy-kidney link might be: retinal microvascular abnormalities resulting from diabetes, hypertension, cigarette smoking, inflammation and other process may provide a common pathophysiologic link for the development and progression of CKD. In our analysis, the prevalence of retinopathy was significantly higher among participants with CKD, compared with participants without CKD, which is consistent with the previous report [11]. Among the two indicators of kidney damage, proteinuria was found to be independently associated retinopathy, which further support that both retinopathy and proteinuria are markers of systematic microvascular abnormalities. Furthermore, the association between proteinuria and retinopathy existed among participants without hypertension or diabetes, suggesting that susceptibility to microvascular disease may be caused by mechanisms other than those directly stemming from hypertension or diabetes.

Age-related macular degeneration has been associated with CKD with varying results. In a cohort study from Austria [15] indicate that participants with eGFR< 60 mL/min/1.73m^2 ^were 3 time more likely to develop early age-related macular degeneration. Another population-based cohort study found that level of serum cystatin C was associated with the incidence of early age-related macular degeneration, while the results for eGFR is not conclusive [7]. A recent cross-sectional study suggested that proteinuria is associated with age-related macular degeneration among men but not among women [8]. Besides age-related macular degeneration, intraocular pressure was also found to be associated with CKD in a population-based cross-sectional study from Singapore [10]. However, no significant association between glaucoma suspect and CKD was observed, possibly due to the small number of participants with glaucoma suspect (4.5%) [10]. In our analysis, the prevalence of both age-related macular degeneration and glaucoma suspect was significantly higher among participants with CKD compared with other participants.

Blindness and low vision are widely recognized as important causes of impairment [16]. Diabetic retinopathy, age-related macular degeneration and glaucoma are important cause of blindness in United States [17], while glaucoma is more common among Chinese population [18]. Our study suggested a high prevalence of those eye abnormalities among participants with CKD, which highlight the importance of eye examination among Chinese CKD patients to improve the overall prognosis of those patients.

Our study has limitations that deserve mention. First, ocular fundus pathology was evaluated by ophthalmoscopes other than photography; and glaucoma suspects were defined only as cup-to-disc ratio of 0.6 or higher. However, all of 3 ophthalmologists have clinical experience of more than 20 years, and they were not aware of participants' clinical conditions. Furthermore, in our Logistic regression models, factors independently associated with eye pathology were consistent with previous reports. However, this type of between-observer misclassification would tend to bias our study towards not finding an association; therefore, it is possible that we underestimated the true association between proteinuria and eye pathology. Second, proteinuria was evaluated by urine dipstick test, which is not as specific as urinary albumin-to-creatinine ratio. However, proteinuria by dipstick was found to be associated other outcomes of CKD including all-cause mortality and cardiovascular mortality [1]. Finally, since our study was observational, the possibility of residual confounding by some unmeasured covariate exists.

## Conclusion

Our study suggests that ocular fundus pathology is common among Chinese patients with CKD. An independent association of ocular fundus pathology with proteinuria was observed; therefore regular eye exam among persons with proteinuria is warranted. That information could be helpful to improve the quality of life and prognosis of CKD patients in China.

## Competing interests

The authors declare that they have no competing interests.

## Authors' contributions

GB and ZL formed the study concept, analyzed the data, interpreted the results, and drafted the manuscript. PY interpreted the results and revised the manuscript. YS collected the data and revised the manuscript. ZL formed the study concept, interpreted the results, and revised the manuscript. WH revised the manuscript for important intellectual content. All authors read and approved the final manuscript.

## Pre-publication history

The pre-publication history for this paper can be accessed here:

http://www.biomedcentral.com/1471-2369/12/62/prepub
